# The Signal Peptide of *Staphylococcus aureus* Panton Valentine Leukocidin LukS Component Mediates Increased Adhesion to Heparan Sulfates

**DOI:** 10.1371/journal.pone.0005042

**Published:** 2009-04-06

**Authors:** Anne Tristan, Yvonne Benito, Roland Montserret, Sandrine Boisset, Eric Dusserre, Francois Penin, Florence Ruggiero, Jerome Etienne, Hugues Lortat-Jacob, Gerard Lina, M. Gabriela Bowden, François Vandenesch

**Affiliations:** 1 Université Lyon 1, Faculté Laennec, Lyon, France; 2 INSERM U851, Centre National de Référence des Staphylocoques, Lyon, France; 3 Hospices Civils de Lyon, Centre de Biologie Est, Lyon, France; 4 Institut de Biologie et Chimie des Protéines, UMR 5086, Université de Lyon, IFR128 BioSciences Gerland-Lyon Sud, Lyon, France; 5 Institut de Biologie Structurale, UMR 5075 CEA-CNRS-UJF, Grenoble, France; 6 Center for Extracellular Matrix Biology, Institute of Biosciences and Technology, The Texas A&M University System Health Science Center, Houston, Texas, United States of America; Baylor College of Medicine, United States of America

## Abstract

*Staphylococcus aureus* necrotizing pneumonia is a severe disease caused by *S. aureus* strains carrying the Panton Valentine leukocidin (PVL) genes (*luk*S-PV & *luk*F-PV) encoded on various bacteriophages (such as phiSLT). Clinical PVL+ strains isolated from necrotizing pneumonia display an increased attachment to matrix molecules (type I and IV collagens and laminin), a phenotype that could play a role in bacterial adhesion to damaged airway epithelium during the early stages of necrotizing pneumonia (J Infect Dis 2004; 190: 1506–15). To investigate the basis of the observed adhesion of *S. aureus* PVL+ strains, we compared the ability of PVL+ and their isogenic PVL− strains to attach to various immobilized matrix molecules. The expression of recombinant fragments of the PVL subunits and the addition of synthetic peptides indicated that the processed LukS-PV signal peptide (LukS-PV SP) was sufficient to significantly enhance the ability of *S. aureus* to attach to extracellular matrix (ECM) components. Furthermore, we showed that adhesion to ECM components was inhibited by heparin and heparan sulfates (HS) suggesting that *in vivo*, HS could function as a molecular bridge between the matrix and *S. aureus* expressing the LukS-PV signal peptide. Site directed mutagenesis, biochemical and structural analyses of the LukS-PV signal peptide indicate that this peptide is present at the *S. aureus* surface, binds to HS in solid phase assay, and mediates the enhanced *S. aureus* matrix component adhesion. Our data suggests that after its cleavage by signal peptidase, the signal peptide is released from the membrane and associates to the cell wall through its unique C-terminus sequence, while its highly positively charged N-terminus is exposed on the bacterial surface, allowing its interaction with extracellular matrix-associated HS. This mechanism may provide a molecular bridge that enhances the attachment of the *S. aureus* PVL+ strains to ECM components exposed at damaged epithelial sites.

## Introduction

Panton-Valentine leukocidin (PVL) is a two-component (LukS-PV and LukF-PV), hetero-oligomeric pore-forming toxin which prevalence in *S. aureus* isolates used to be low (ca. 2%) but is currently increasing due to the worldwide diffusion of PVL-producing methicillin-resistant *S. aureus* (MRSA) strains [1]. PVL is associated with staphylococcal strains that cause deep skin and soft tissue infections as well as severe necrotizing pneumonia [2]. Necrotizing pneumonia affects children and young adults (median age 15 years) [3], is often preceded by a viral-like illness, and is characterized as a rapidly expansive pneumonia, associated with haemoptysis, leukopenia and pleural effusion that can progress towards acute respiratory distress syndrome [3], [4]. Lung tissues from staphylococcal necrotizing pneumonia patients revealed that respiratory epithelium ulcerations that extend from the larynx to the lobar bronchi were heavily colonized with abundant Gram-positive *cocci* [3]. *In vitro*, PVL+ clinical isolates from necrotizing pneumonia display an increased adhesion to extracellular matrix molecules (ECM) present in epithelial basement membranes and in the subjacent connective tissue such as collagen I, IV and laminin [5]. The enhanced attachment to damaged airway epithelium could play a central role during bacterial colonization and the early stages of infection.

To investigate the basis of the observed increased adhesion of *S. aureus* PVL+ strains, we compared the ability of isogenic PVL+ and PVL− strains to attach to various immobilized matrix molecules. The expression of fragments of the PVL subunits, along with the addition of synthetic peptides indicated that the LukS-PV signal peptide (LukS-PV SP) is sufficient to significantly enhance the ability of *S. aureus* to attach to matrix components. Moreover, we show here that adhesion to ECM components is efficiently inhibited by heparin/heparan sulfates (HS) suggesting that *in vivo*, HS could function as bridges between *S. aureus* expressing the LukS-PV signal peptide and ECM components exposed after epithelium damage. The biochemical properties of the LukS-PV signal peptide indicate that it could be released outside the cytoplasmic membrane after signal peptidase cleavage, which was confirmed by the use of antibodies directed against an histidine-tagged LukS-PV signal peptide. Site directed mutagenesis of the LukS-PV signal peptide supports the hypothesis that the C-terminus of the signal peptide is associated to the bacterial cell wall and the N-terminus, which is highly positively charged, binds to matrix-associated heparan sulfates.

## Results

### Expression of the *luk*-PV operon increases the adhesion of *S. aureus* and *Lactococcus lactis* to various ECM proteins

**Figure 1:**
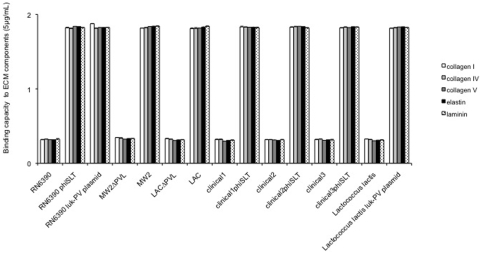
Binding capacity of isogenic strains from various genetic backgrounds to ECM components. Isogenic strains of *Staphylococcus aureus* and *Lactococcus lactis* examined for their binding capacity (Y-axis, absorbance reading at 540 nm) to ECM proteins (Sigma) coated on 96-well plates, at concentrations of 5 µg/mL (identical results were obtained at concentrations of 50 µg/mL). Collagen I (white bars); collagen IV (grey bars); collagen V (dark grey bars); elastin (black bars), laminin (dotted bars). RN6390, ref agr1 strain, *luk*-PV negative; RN6390phiSLT, RN6390 lysogenized with phiSLT; RN6390 Luk-PV plasmid, RN6390 carrying a *luk*-PV plasmid; MW2ΔPVL, MW2 deleted of the *luk*-PV operon; MW2 wild type (*luk*-PV positive); LACΔPVL, LAC deleted of the *luk*-PV operon; LAC, LAC wild type (*luk*-PV positive); clinical1, *luk*-PV negative clinical strain from gut; clinical1phiSLT, clinical 1 lysogenized with phiSLT; clinical2, *luk*-PV negative clinical strain from furuncle; clinical2phiSLT, clinical2 lysogenized with phiSLT; clinical3, *luk*-PV negative clinical strain from pneumonia; clinical3phiSLT, clinical3 lysogenized with phiSLT; *Lactococcus lactis*, *L. lactis* ; *Lactococcus lactis* Luk-PV plasmid, *L. lactis* carrying a *luk*-PV plasmid. The vertical lines indicate the standard deviations.

To determine if the enhanced attachment of the clinical PVL-positive isolates to ECM proteins was mediated by gene products encoded by the phage that carries PVL (phiSLT), by PVL itself, or by other genes present in the *S. aureus* chromosome, we constructed a series of isogenic strains lysogenizing the phage phiSLT into several PVL negative clinical isolates. We also included isogenic strains made by allelic replacement of the *luk*-PV genes (*luk*S-PV and *luk*F-PV) in PVL positive strains. All these strains were tested for their ability to attach to various immobilized extracellular matrix proteins: collagen IV and laminin, representing basement membrane components, and collagens V, I and elastin, all present in the subjacent lung connective tissue (Figure 1). Compared to the PVL negative strains (RN6390, clinical 1, 2 and 3) the lysogenized strains (RN6390 phiSLT, clinical 1, 2 and 3 phiSLT) displayed a significantly higher (>6 fold) adhesion to all the ECM components tested, with a level of adhesion similar to those mediated by MSCRAMMs [6]. Similarly, compared to the natural PVL+ strains MW2 and LAC, the delta-*luk*-PV derivatives (MW2ΔPVL, LACΔPVL) adhered poorly to ECM proteins. Thus, the enhanced attachment phenotype previously observed in non-isogenic clinical isolates, was confirmed to be associated with the expression of an intact PVL operon. To confirm this hypothesis, we transformed *S. aureus* RN6390 with a plasmid expressing the *luk*-PV operon in trans (Luk-PV plasmid) and tested the ability of the resulting strain to attach to ECM proteins. The results revealed that the *luk*-PV operon was sufficient to confer *S. aureus* the enhanced adhesion to ECM proteins (Figure 1). To test if this property could be transferred in an heterologous host, the Luk-PV plasmid was introduced into *Lactococcus lactis*; indeed, the resulting strains showed enhanced adhesion similar to that of all strains of *S. aureus* expressing an intact PVL.

### The LukS-PV signal peptide mediates the enhanced attachment (Figure 2)

**Figure 2:**
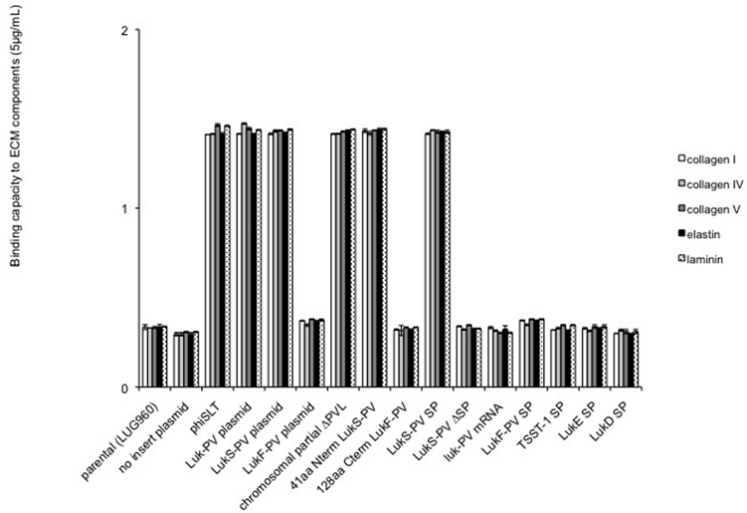
Binding capacity of isogenic strains to ECM components. Isogenic strains of *Staphylococcus aureus* examined for their binding capacity (Y-axis, absorbance reading at 540 nm) to collagen IV (Sigma) coated on 96-well plates, at concentrations of 5 µg/mL (identical results were obtained at concentrations of 50 µg/mL). Collagen I (white bars); collagen IV (grey bars); collagen V (dark grey bars); elastin (black bars), laminin (dotted bars). Parental (LUG960) indicates the genetic background in which all genetic modifications were made as follows: no insert plasmid, plasmid vector with no insert; phiSLT, lysogenization with phiSLT; Luk-PV plasmid, plasmid encoding for *luk*S-PV and *luk*F-PV; LukS-PV plasmid, plasmid encoding for *lukS*-PV; LukF-PV, plasmid encoding *lukF*-PV; chromosomal partial ΔPVL, partial deletion of the chromosomal *luk*-PV operon; 41aa Nterm LukS-PV, plasmid encoding for the first 41 amino acids of premature LukS-PV; 128aa Cterm LukF-PV, plasmid encoding for the last 128 amino acids of premature LukF-PV; LukS-PV SP, plasmid encoding the LukS-PV signal peptide; LukS-PV ΔSP, plasmid encoding LukS-PV without signal peptide; *luk*-PV mRNA, plasmid encoding for the *luk*-PV operon mRNA devoid of start codon; LukF-PV SP, plasmid encoding the LukF-PV signal peptide; TSST-1 SP, plasmid encoding the TSST-1 signal peptide; LukE-SP, plasmid encoding for the LukE signal peptide; LukD-SP; plasmid encoding for the LukD signal peptide. The vertical lines indicate the standard deviation.

To establish which gene of the *luk*-PV operon (*luk*S-PV or *luk*F-PV) was required to induce this phenotype, *luk*S-PV and *luk*F-PV genes were cloned and expressed independently in *S. aureus* RN6390 delta-*spa* (LUG960); this strain was chosen to avoid any possible effects mediated by an increased transcription of Spa [7]. Compared to the parental strain (LUG960), or to the strain expressing the plasmid with no insert (no insert plasmid), the expression of LukS-PV alone (LukS-PV plasmid) resulted in enhanced adhesion phenotype similar to that induced by the complete operon (Luk-PV plasmid) (Figure 2). In contrast, expression of LukF-PV alone did not result in a significant enhanced adhesion phenotype. A major chromosomal deletion of the *luk*-PV operon in LUG960 preserving the 41 N-terminal amino acids of pre-mature LukS-PV and 128 C-terminal amino acids of LukF-PV (chromosomal partial ΔPVL) showed an attachment phenotype similar to the strain that carries an intact *luk*-PV operon (Figure 2). We thus cloned and expressed either the 41 N-terminal aa of pre-mature LukS-PV or the 128 C-terminal amino acids of LukF-PV in LUG960 cells under the control of the P-*luk*-PV promoter. Compared to the parental strain, the strain expressing the first 41 amino acids of pre-mature LukS-PV showed an enhanced adhesion phenotype similar to that of the strain expressing the intact Luk-PV operon. In contrast, the strain expressing the 128 C-terminal amino acids of LukF-PV had no effect on adhesion. Further subcloning revealed that the sequence encoding the 28 amino acids signal peptide of LukS-PV (LukS-PV SP) was sufficient to mediate enhanced bacterial attachment to ECM components. Conversely, the strain LukS-PV ΔSP expressing mature LukS-PV devoid of its signal peptide showed a basal level of attachment, similar to that shown by a strain carrying the vector without insert. To rule out the possibility that the mRNA encoding the LukS-PV signal peptide but not the signal peptide itself was involved, a construct expressing *luk*-PV mRNA devoid of its start codon was expressed in LUG960 (a control RT-PCR experiment revealed that the construct produced the corresponding mRNA, data not shown). This *luk*-PV mRNA construct did not increase attachment to ECM proteins. These data confirms that the expression of the 28 amino acids constituting the LukS-PV signal peptide mediate the enhanced attachment phenotype.

### The enhanced attachment phenotype is specific to the LukS-PV signal peptide (Figure 2)

To determine if the phenotype observed with LukS-PV signal peptide could be a general property of other staphylococcal signal peptides, we expressed the LukF-PV signal peptide, the TSST-1 signal peptide (unrelated to Luk-PV SP), the LukE signal peptide (64% similarity to LukS-PV signal peptide) and the LukD signal peptide (unrelated to LukS-PV and 87% similarity to LukF-PV signal peptide) under the control of the *luk*-PV promoter. Figure 2 showed that only the expression of LukS-PV signal peptide can mediate the enhanced bacterial attachment to ECM proteins. A similar enhanced attachment to ECM proteins was obtained when expressing the full-length LukS-PV, LukS-PV signal peptide in *Lactococcus lactis*, and not when expressing LukE signal peptide (data not shown).

### Adhesion to matrix proteins could be mediated by heparan sulfates (HS)

**Figure 3:**
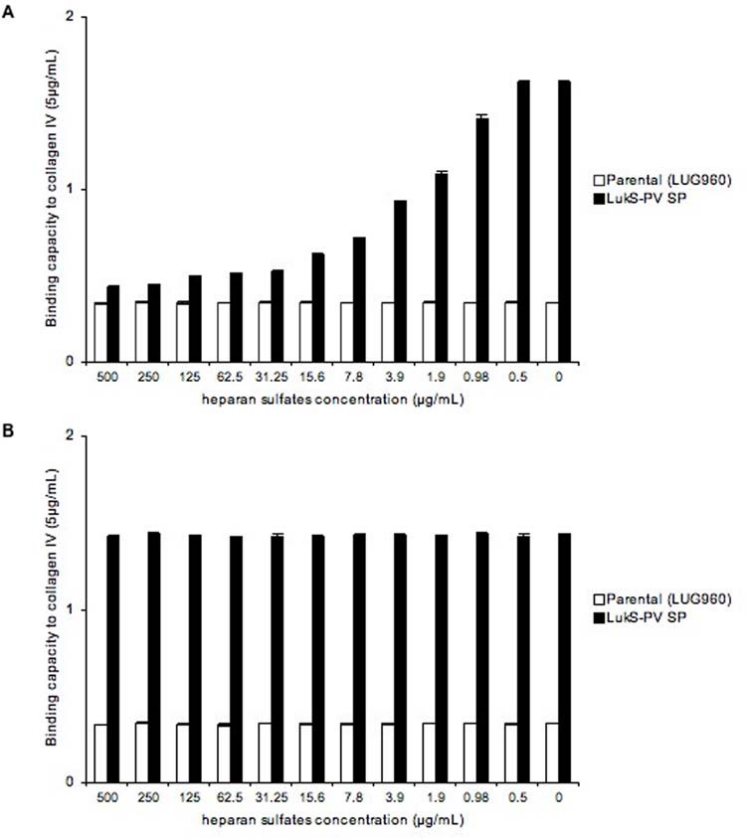
Inhibition of adhesion to collagen IV by heparan sulfates. Isogenic strains of *Staphylococcus aureus* examined for their binding capacity (Y-axis, absorbance reading at 540 nm) to collagen IV (Sigma) coated on 96-well plates, at concentrations of 5 µg/mL. Heparan sulfates were added at decreasing concentrations (X-axis) to the bacteria before the adhesion assay (panel A), or after the preincubation of the bacteria with collagen IV (Sigma) (panel B). Parental (LUG960) indicates the genetic background in which the genetic modifications were made; LukS-PV SP, parental carrying a plasmid encoding the LukS-PV signal peptide. The vertical lines indicate the standard deviation.

**Figure 4:**
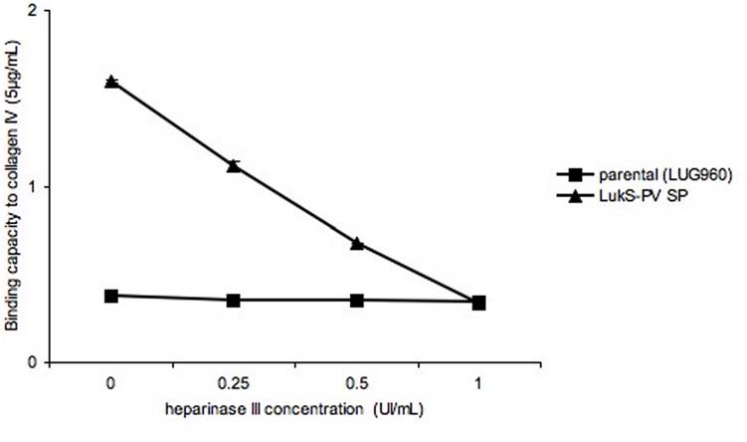
Inhibition of adhesion to collagen IV by heparinase. Isogenic strains of *Staphylococcus aureus* examined for their binding capacity (Y-axis, absorbance reading at 540 nm) to collagen IV (Sigma) coated on 96-well plates, at concentrations of 5 µg/mL. Heparinase III was added at increasing concentrations (X-axis) to collagen IV (Sigma) before the adhesion assay. Parental (LUG960) indicates the genetic background in which the genetic modifications were made; LukS-PV SP, parental carrying a plasmid encoding the LukS-PV signal peptide. The vertical lines indicate the standard deviations.

**Figure 5:**
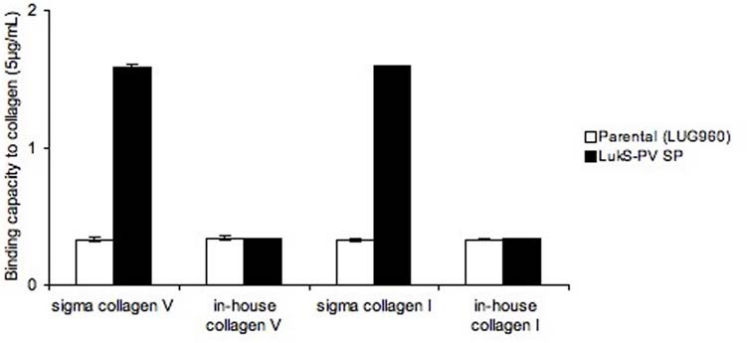
Binding capacity of isogenic strains to collagens V and I from distinct sources. Isogenic strains examined for their binding capacity (Y-axis, absorbance reading at 540 nm) to collagens I and V (Sigma) *versus* in-house preparation of collagens I and V coated on 96-well plates, at concentrations of 5 µg/mL. Parental (LUG960) indicates the genetic background in which the genetic modifications were made; LukS-PV SP, parental carrying a plasmid encoding the LukS-PV signal peptide. The vertical lines indicate the standard deviations.

**Figure 6:**
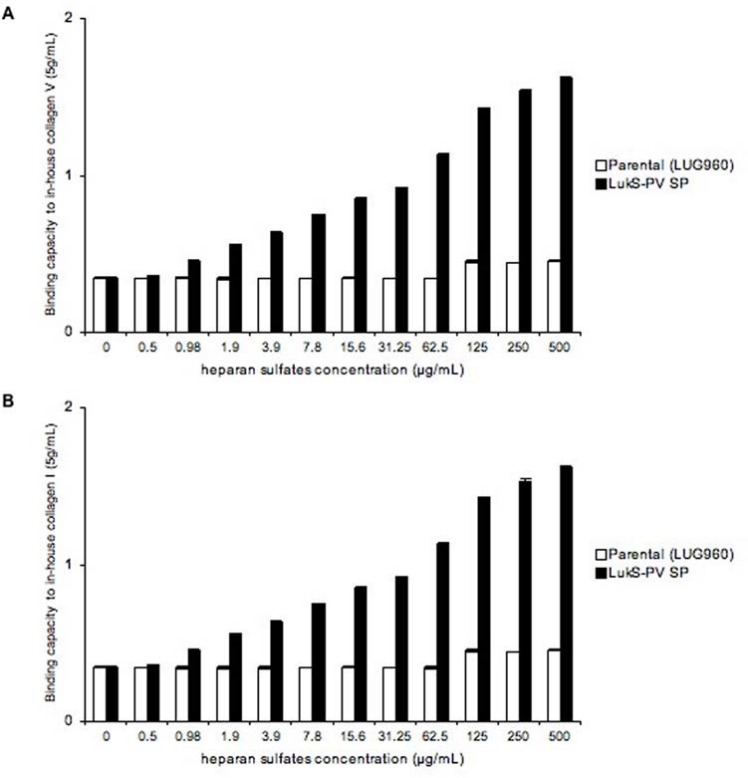
Binding capacity of isogenic strains to in-house collagens V and I after addition of increasing concentrations of heparan sulfates. Isogenic strains examined for their binding capacity (Y-axis, absorbance reading at 540 nm) to collagens I and V preparations from our laboratory coated on 96-well plates, at concentrations of 5 µg/mL. Before the adhesion assay, heparan sulfates were added at increasing concentrations (X-axis) to the plates coated with recombinant collagen V (panel A) or highly purified collagen I (panel B). Parental (LUG960) indicates the genetic background in which the genetic modifications were made; LukS-PV SP, parental carrying a plasmid encoding the LukS-PV signal peptide. The vertical lines indicate the standard deviations.

Since the various ECM proteins tested in the present work yielded similar results, a possible common mechanism was sought. We reasoned that it would be unlikely to find a common structural motif among all tested ECM proteins (collagens, laminins and elastin) that could serve as a ligand for the LukS-PV-signal peptide-expressing strains. However, we hypothesized that heparan sulfates (HS) could be the ligand of the LukS-PV signal peptide-expressing strain, since HS are known to bind several ECM proteins and could be present in trace amount in commercially available purified preparations of ECM proteins (Sigma-Aldrich technical service does not guarantee their ECM preparations as free of heparan sulfates). Due to their high negative charge, HS chains interact with a multitude of proteins, including growth factors/morphogens and their receptors, chemokines, and extracellular-matrix proteins [8]. To test this hypothesis, we used heparin and heparan sulfates (HS) as soluble competitors in the immobilized ECM adhesion assay. Preincubation of the bacteria with increasing concentrations of HS reduced the adhesion to all matrix molecules tested (Figure 3A and Figure S1). Similar results were obtained when using heparin instead of heparan sulfates (data not shown). However, preincubation of the various matrix proteins with HS prior to the addition of bacteria did not alter the bacterial adhesive properties, nor did the preincubation of bacteria with matrix proteins followed by the addition of HS (Figure 3B). When increasing concentrations (X-axis) of heparinase III was added to the matrix protein prior to the adhesion assay, the bacterial adhesion decreased in a dose-dependent manner (Figure 4). In a converse experiment, we used collagen I and V from distinct sources. Collagen I was extracted from bovine bones using pepsin and recombinant V was produced in the human embryonic kidney cell line (293-HEK) cells (see Material and Methods). These preparations are thought to be both devoid of linked heparan sulfates. A strain expressing LukS-PV signal peptide did not show enhanced adhesion to collagens I and V purified in our laboratory, while it did when using collagen I and V purchased from Sigma (Figure 5). Interestingly, preincubation of the in-house preparations of collagen I and V with heparan sulfates, followed by the addition of bacteria, enhanced the staphylococcal adhesion to both substrates (Figure 6). Collagens I and V are known to contain a heparin binding site [9], [10] and are good candidates for HS-mediated cell interactions. Taken together, these data suggest that the ligand of LukS-PV signal peptide-expressing strains are heparan sulfates.

### Validation of the interaction between LukS-PV signal peptide and HS

**Figure 7:**
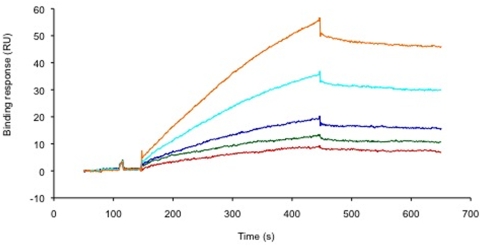
The LukS-PV signal peptide binds to heparan sulfates in a solid phase assay. Overlay of sensorgrams obtained when LukS-PV signal peptide at (from top to bottom) 45, 30, 20, 13.3 and 8.9 µg/ml was injected for 5 minutes at 50 µl/min over a HS activated sensorchip. The Binding response (in RU) was recorded as a function of time (in s).

To determine the HS binding ability of the LukS-PV signal peptide, we used a solid phase assay, where the reducing end of biotinylated HS was captured on a streptavidin coated sensorchip. This system mimics, to some extent, the cell surface- or the extracellular matrix-anchored proteoglycans. Surface plasmon resonance (SPR) real time monitoring was performed to measure changes in refractive index of the chip after the binding of the peptide to the immobilized HS. Injection of a range of concentrations of LukS-PV signal peptide over the HS-coated sensorchip is represented by the binding curves shown in Figure 7, whereas similar injections over a control surface (containing streptavidin only) did not lead to significant signal (data not shown), demonstrating the existence of an interaction between the polysaccharide and the peptide. Analysis of these sensorgrams, using a simple A+B = AB model (single binding site model), indicates an affinity in the low µM range (Kd = 5.6±2.2 µM). Presumably, at the bacterial surface, the multiplicity of the LukS-PV signal peptide should strongly contribute to avidity, resulting in tighter interactions.

### Extracellular LukS-PV SP mediates bacterial attachment

**Figure 8:**
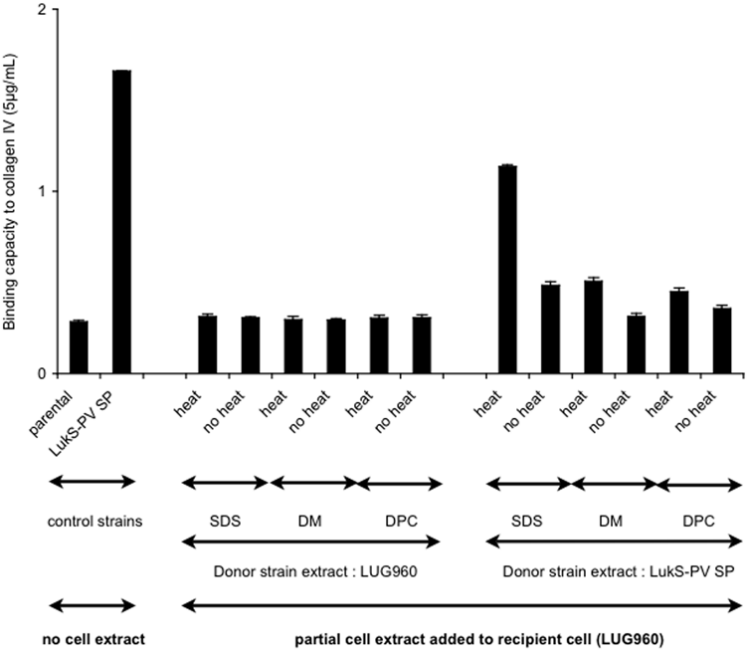
Adherence is mediated by cell wall associated material. Following overnight culture and centrifugation, the bacterial pellet of the donor strain (parental or parental carrying a plasmid expressing LukS-PV signal peptide) was resuspended in 1 mL extraction buffer (2% SDS or DM or DPC), heated (or not) at 95°C for 2 min, and centrifuged. The supernatants were used to resuspend the bacterial pellet of the recipient strain to be tested (parental) in the adherence assay to type IV collagen (5 µg/mL, Sigma) coated on 96-well plates. The first two lanes are control strains (parental and parental expressing LukS-PV signal peptide) with no cell extract added. All other lanes indicate the various conditions of cell extracts added to the parental. The vertical lines indicate the standard deviations.

**Figure 9:**
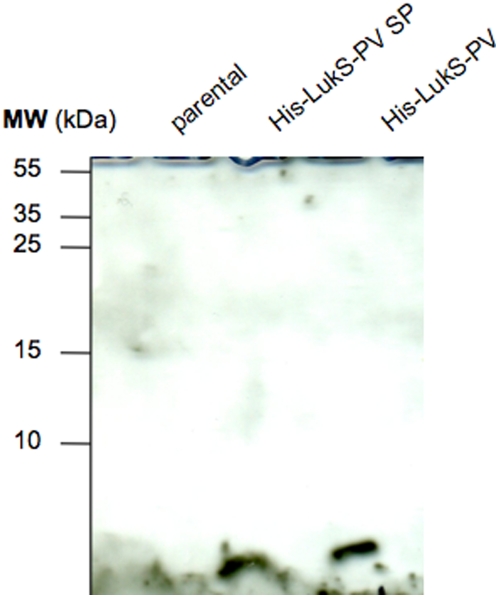
The signal peptide is present in cell wall associated material. The 2% SDS extract of various strains were run on 17% SDS PAGE, transferred to nylon membrane and probed with mouse monoclonal anti-poly His antibody (1∶500) (Sigma) followed by goat anti mouse HRP conjugated antibody (1∶2000) (Sigma). Parental (LUG960) indicates the genetic background in which the genetic modifications were made; His-LukS-PV SP, parental carrying a plasmid encoding the His-tagged LukS-PV signal peptide alone; His-LukS-PV, parental carrying a plasmid encoding the His-tagged full length LukS-PV.

To test if the attachment of the LukS-PV signal peptide-expressing strain was mediated by cell-surface-associated materials, extracellular detergent extracts of the LukS-PV signal peptide-expressing (donor) strain were prepared using either 2% SDS (sodium dodecylsulfate), 2% DPC (dodecylphosphocholine), or 2% DM (dodecylmaltoside). These extracts were then added to LUG960 (recipient) cells; the treated bacteria were then tested for their capacity to adhere to collagen IV from commercial source (Sigma-Aldrich). Incubation of the 2% SDS extracellular extract from the donor strain with the recipient strain resulted in enhanced adhesion to collagen IV (Figure 8). In addition, the addition of extracellular extracts obtained with mild detergents (DM or DPC) to the recipient cells resulted in a modest enhanced adhesion (Figure 8). Conversely, no increased adhesion was observed when using extracellular cell extracts (regardless of the detergent used) from LUG960 used as donor cells (Figure 8). These data indicate that a SDS soluble compound present at the surface of LukS-PV positive strains is able to confer the enhanced adhesion phenotype. To determine if the LukS-PV SP was present in this extract, a 2% SDS extract was prepared from an *S. aureus* strain expressing an His-tag LukS-PV signal peptide fusion or a His-tag LukS-PV fusion. Western blot analysis performed using an anti-His-tag antibody showed in both extracts a band of the expected size (approximately 3 kDa) (Figure 9), demonstrating that the signal peptide was present in the SDS extracts. This Figure also shows that unprocessed His-tagged LukS-PV was not detected in the western blot, demonstrating the efficiency of the signal peptide processing. In addition, control experiments revealed that the secretory function of the signal peptide was not impaired by the His-tag, since the mature LukS-PV component of the PVL was detected by ELISA in the bacterial supernatant (Table S1).

**Figure 10:**
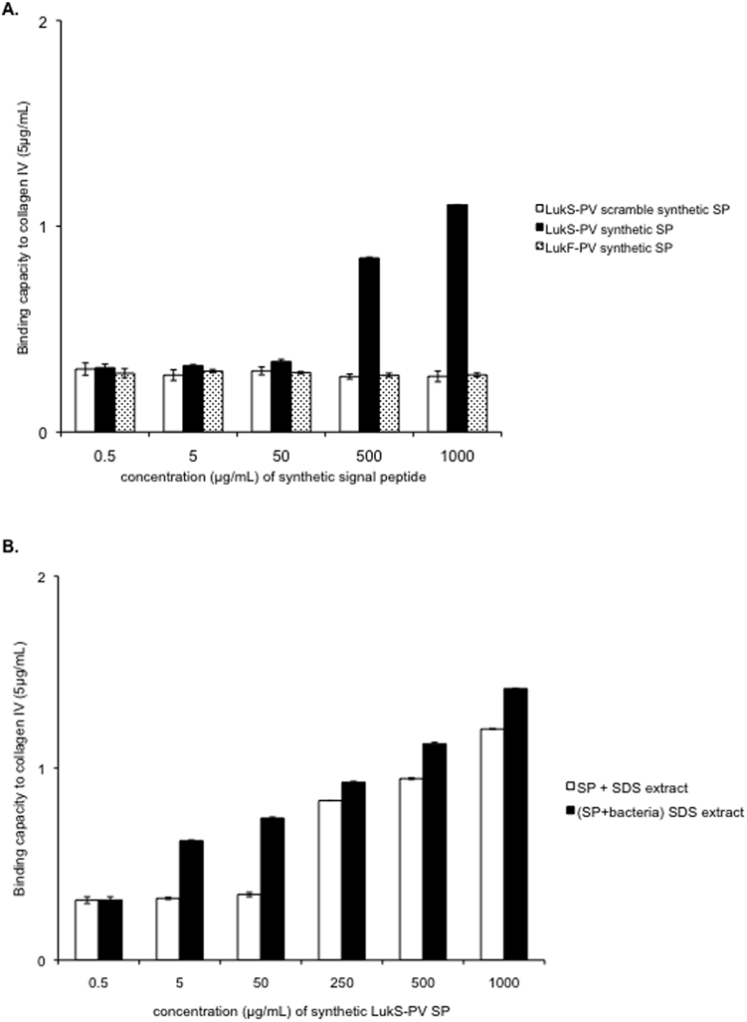
Effect of the addition of synthetic signal peptide on the adherence capacity of the parental strain. A. Increasing concentrations of synthetic LukS-PV signal peptide, LukF-PV signal peptide and scrambled synthetic LukS-PV signal peptide were added to parental cells (LUG960); the mixtures were then tested for adhesion to type IV collagen (Sigma, 5 µg/mL) coated on 96-well plates. The vertical lines indicate the standard deviations. B. Increasing concentrations of synthetic LukS-PV signal peptide was added to the 2% SDS extract of the parental strain (white bars, SP+SDS extract) or to the parental strain before 2% SDS extraction (black bars, (SP+Bacteria) SDS extract); in both cases the mixture was used to resuspend the bacterial pellet of the recipient strain (parental) in the adherence assay to type IV collagen (5 µg/mL, Sigma) coated on 96-well plates.

To test whether the signal peptide could on its own enhance bacterial attachment, synthetic peptides corresponding to LukS-PV signal peptide, LukF-PV signal peptide, and a scrambled version of the LukS-PV signal peptide were added to the parental bacterial strain (LUG960) in the microtiter adhesion assay. External addition of the synthetic LukS-PV signal peptide, but not the synthetic LukF-PV signal peptide nor the scrambled LukS-PV signal peptide resulted in a dose-dependent enhanced adhesion (Figure 10 A). This reveals that the signal peptide of LukS-PV contributes to the enhanced adhesion phenotype of PVL-positive strains. Enhanced adhesion was observed only at synthetic peptide concentrations above 500 µg/ml; however, when a 2% SDS extract was prepared from non-adhering bacterial cells plus the synthetic peptide, the resulting preparation conferred increased adhesion at lower synthetic peptide concentrations (Figure 10B). This suggests that the concomitant presence of an unknown cell-wall component with the synthetic signal peptide is required in order to confer the full adhesion phenotype. However, we cannot exclude that some post-translational modification may occur in the physiological situation, thus explaining why the synthetic peptide remains less efficient to restore adhesion than that produced *in vivo*.

### Conformational analysis of LukS-PV SP and LukF-PV SP by circular dichroism (CD)

**Figure 11:**
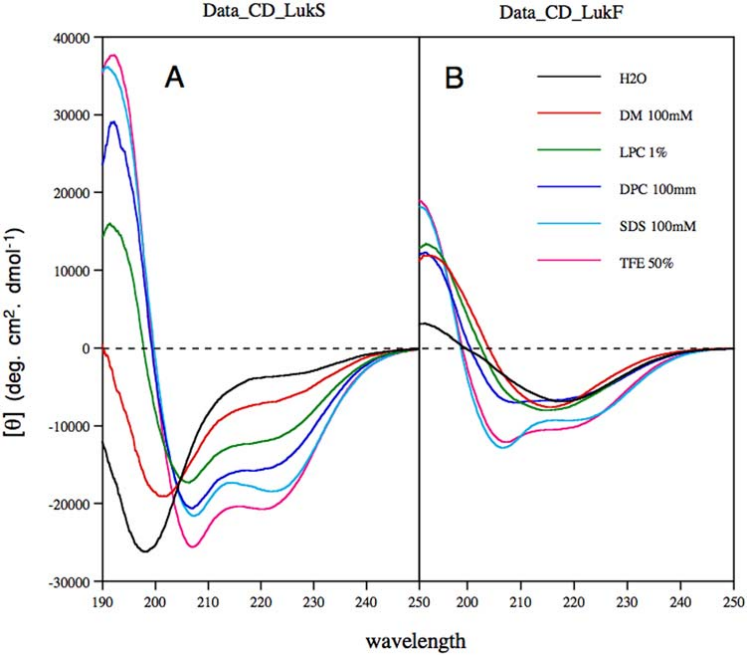
Far-UV circular dichroism (CD) analyses of synthetic peptides LukS-PV SP (A) and LukF-PV SP (B) in various environments. CD spectra were recorded in water (H_2_O), complemented with either 50% 2,2,2-trifluoroethanol (TFE) or 1% L-α-lysophosphatidyl choline (LPC), or the following detergents: 100 mM sodium dodecyl sulfate (SDS), 100 mM n-dodecyl-β-D-maltoside (DM), or 100 mM dodecyl phosphocholine (DPC).

To gain insight into the structure and lipotropic properties of the LukS-PV and LukF-PV signal peptides, the solubility and conformation of the corresponding purified synthetic peptides were examined under various stabilizing conditions by using either cosolvents (TFE: water mixtures, SDS) that are very useful tools for probing the propensity of peptides for secondary structure formation ([11], [12], and references therein), or membrane mimetic media including lysolipid (lysophosphatidyl choline, LPC), and detergents (DM, LPC and DPC). As is expected, both LukS-PV and LukF-PV signal peptides solubilized in SDS exhibited the typical spectra of α-helix folding, with two minima at 208 and 222 nm and a maximum at 192 nm (Figure 11 A–B, cyan curve). TFE is known to solubilize hydrophobic peptides as monomers and to stabilize the folding of peptidic sequences with an intrinsic propensity to adopt an α-helical structure. Dissolving both peptides in 50% TFE generated spectra that were typical of α-helical folding, and similar in intensity to that obtained in SDS (Figure 11 A–B, magenta curve). Various CD deconvolution methods used indicate a predominant α-helix content ranging from 64 to 77% in SDS and in TFE 50% for LukS-PV signal peptide, but only of about 40% for LukF-PV signal peptide in both media. In LPC (Figure 11 B, green curve) and DM (Figure 11 B, red curve), the latter peptide exhibited CD spectra that are similar of ß-sheet-like conformation with a maximum around 190 nm and a minimum around 215 nm, while the shape of CD spectrum recorded in DPC (Figure 11 B, blue curve) suggested a mixture of α-helix and ß-sheet like conformation. In addition, the LukF-PV signal peptide was poorly soluble in water and exhibited a ß-sheet-like conformation indicating of soluble, micelle-like aggregates (Figure 11B, black line). In addition, the LukF-PV signal peptide aggregates over time in the absence of a hydrophobic, membrane-like environment. These findings are consistent with the computer prediction that this peptide could easily aggregate (score of 0.8 with Zyggregator [13]). In contrast, the LukS-PV signal peptide is largely soluble in water, does not exhibit the propensity to aggregate (in keeping with a score of −0.1 with Zyggregator), and exhibits a typical random coil CD spectrum, with a single negative peak at 198 nm (Figure 11A, black line). When studied by NMR, the LukS-PV signal peptide 1D spectrum displays fine and well-resolved signals, indicating that the peptide exists as a monomer in water (data not shown). Moreover, it only partly folds into α-helix in the presence of membrane mimetics (LPC, DPC and DM, Figure 11A). These analyses suggest that the LukS-PV signal peptide has little propensity to bind to the membrane hydrophobic core. Taken together, these analyses indicate that after its processing by the signal peptidase, the LukS-PV signal peptide could readily leave the membrane translocon context and incorporate to the cytosolic or the extracellular aqueous environment.

### Identification of LukS-PV signal peptide residues involved in the adhesion phenotype using mutagenesis

The above data favor a model in which the LukS-PV signal peptide after cleavage by the signal peptidase is released outside the cytoplasmic membrane but remains associated to the cell surface and confers adhesion to heparan sulfates. Given the amino acid composition of the peptide and the homologies with other signal peptides that do not confer increased adhesion (especially LukE signal peptide which is the most closely related), we hypothesized that the C-terminus of LukS-PV signal peptide could be bound to the cell wall and the highly positively charged N-terminus could bind to negatively charged heparan sulfates. To test this hypothesis, a series of deletions and point mutations of the LukS-PV signal peptide were constructed, as well as chimeras combining portions of LukS-PV signal peptide and LukE signal peptide.

**Figure 12:**
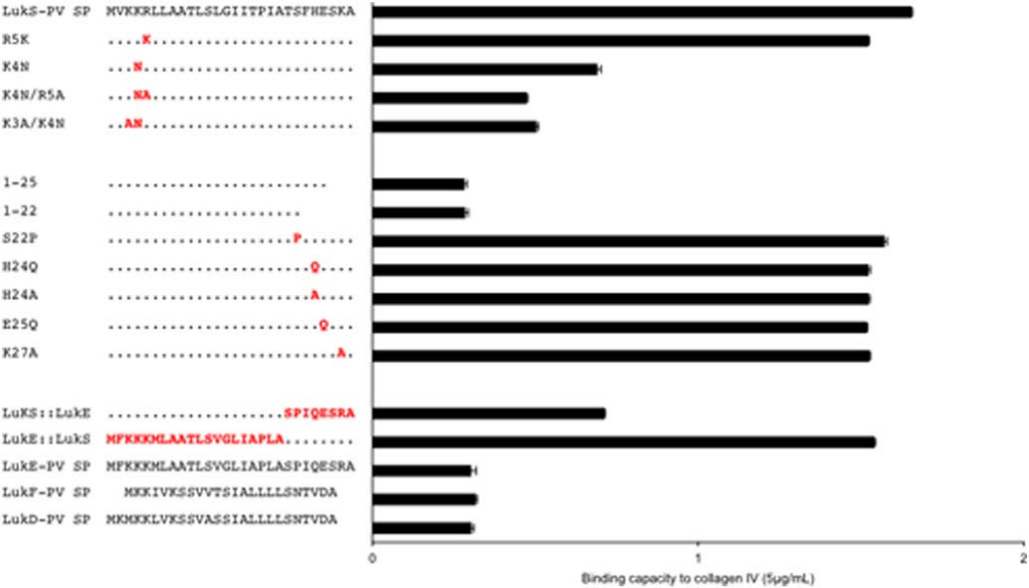
Identification of LukS-PV signal peptide residues involved in the adhesion phenotype using mutagenesis. Sequences of wild type LukS-PV signal peptide (top) and mutants are shown on the left side, together with the alignment of sequences of LukE, LukF-PV and LukD signal peptides (bottom). The binding capacity of the corresponding sequences to type IV collagen (5 µg/mL, Sigma) is shown on the right side. Mutated amino acids are colored red while other LukS-PV SP amino acids are represented by dots. Deletion mutants of LukS-PV SP are denoted by their sequence length (1–25 and 1–22).

On the N-terminus, a single residue substitution that replaced a positively charged residue with another one (R5K) did not alter the adhesion property of the resulting peptide. In contrast, replacing positively charged residue with neutral one (K4N) significantly reduced adhesion to collagen IV, whilst the double substitutions K4N/K5A and K3A/K4N reduced the adhesion even more (Figure 12). These data highlight the strict requirement of three positives charges on the N-terminus for efficient adhesion.

A LukS-PV signal peptide deletion mutant in which the last 6 (1–22) or only 3 (1–25) C-terminal residues were removed, completely abolished the adhesion property of the peptide-expressing strains, revealing that a full-length C-terminus is required for adhesion. Single amino acid substitutions of the LukS-PV peptide C-terminus with residues biochemically similar to those present in the LukE signal peptide (S22P or H24Q) or with residues of different charge properties (H24A, E25Q or K27A) did not abolish adhesion. Finally, a strain expressing a chimera composed of the N-terminal half of the LukE signal peptide fused to the C-terminal half of LukS-PV signal peptide exhibited the full adhesion phenotype, whilst the reverse chimera (LukS-PV SP::LukE SP) did not enhance adhesion (Figure 12). To ensure that the modified peptides properly function as signal peptides, several of the above-described mutated peptides were expressed in fusion with the LukS-PV component of PVL that they were supposed to secrete. Quantification of LukS-PV in the supernatant confirmed that the selected mutations in the signal peptide did not affect the efficiency of the secretion machinery. Conversely, when LukS-PV PVL was expressed without signal peptide, only residual amount of LukS-PV was detected in the supernatant (Table S1). Altogether, these data indicate that the positively charged amino acid cluster of the N-term interacts with heparan sulfates and that a stretch of C-terminal residues unique to the LukS-PV-signal peptide (TSFHESKA) likely constitute an important determinant for the peptide association to the bacterial cell wall. This C-terminal sequence can tolerate single amino acid substitutions, suggesting the existence of a rather wide binding region for interaction with cell wall components.

## Discussion

Clinical PVL+ *S. aureus* isolates have been reported to display an enhanced attachment to ECM proteins (type I collagen, type IV collagen, and laminin) that are abundant in the lung [5]. During the early stages of infection, ECM proteins from the epithelial basement membrane and the adjacent connective tissue are exposed to damaged epithelium sites. The results obtained with various isogenic strains made in various genetic backgrounds including an heterologous host (*Lactococcus lactis*) confirmed that PVL-positive strains have enhanced attachment to the ECM components, the actual target being heparan sulfates and not directly ECM proteins. Cloning and expression of recombinant fragments of PVL subunits, along with the addition of synthetic peptides indicated that the processed LukS-PV signal peptide, which may act in conjunction with some other cell-wall components, was sufficient to significantly enhance the ability of *S. aureus* to attach to ECM components. Site directed mutagenesis studies of the LukS-PV signal peptide together with the biochemical analyses of the corresponding synthetic peptide supports the following model: after its cleavage by signal peptidase, the signal peptide is released from the membrane and binds to the cell wall through its unique C-terminus sequence (TSFHESKA), while its highly positively charged N-terminus binds to extracellular matrix-associated heparan sulfates. These events would result in a molecular bridge involving the signal peptide and heparan sulfates that would allow the bacteria to attach to the host tissue.

Small peptides are widely used for bacterial communication systems in Gram-positive bacteria [14] but the post-secretory function of signal peptides has not been extensively characterized in prokaryotes. For instance, the *Staphylococcus haemolyticus* hemolysin H7 is made of three 44-amino-acid peptides, each having a structure that resembles a signal peptide, suggesting that these peptides are signal sequences from secreted or membrane-associated proteins [15]. More recently, Firth *et al.* described a lipoprotein signal peptide encoded by the staphylococcal conjugative plasmid pSK41 exhibiting an activity resembling that of *Enterococcus faecalis* pheromone cAD1, which induces clumping of bacterial cells [16]. In contrast, eukaryote signal peptides have been shown as good candidates for additional functions beyond protein targeting to translocon [17]. For instance, the HLA-E epitopes generated from the signal sequence of the polymorphic MHC class I molecule, are further processed and loaded to a non-polymorphic MHC class I molecule HLA-E, generating a complex that is subsequently transported to the cell surface for presentation to natural killer cells [18]. Another example of the multiple functions of signal peptides is the fate of signal peptide fragments of HIV-1 gp160 and of the human preprolactin, which interfere with cytosolic calmodulin, a major protein involved in the regulation of calcium metabolism [17], [19], [20]. As stated by Martoglio, “there are almost undoubtedly other components that interact with signal peptide fragments in the cytosol or the extracellular space that are yet to be discovered” [20]. Despite fundamental differences in cell biology, prokaryote and eukaryote signal peptides harbor similar structure and basic functions, ensuring the proper targeting to the translocation machinery; therefore, the discovery of post-secretory functions for bacterial signal peptides should be expected. In bacteria, nascent precursor proteins associated to ribosomes are targeted to the translocation machinery to cross the membrane. They are then released from the signal peptide by signal peptidase and the remaining signal peptide is generally believed to be degraded by membrane proteases. However, the fate of the signal peptide after processing has not been elucidated [21].

Typical signal peptides are short sequences of 15 to 30 amino acids composed of an N-terminal region (n-domain) including positively charged residues, a hydrophobic core region (h-domain) forming an α-helix, and a more polar, flexible region (c-domain) containing the signal peptidase cleavage site [22]. Despite these common features, experimental analyses of isolated signal peptides revealed various conformational features, as both α-helical and ß-sheet structures have been observed in different media ([23] and references therein). It is thus reasonable to postulate that after the signal peptidase cleavage, the fate of the released signal peptide is likely dependent on their physico-chemical properties. In contrast to the LukF-PV signal peptide, which is poorly soluble and aggregates in water, the LukS-PV signal peptide was shown here to be unfolded and soluble in water, and to exhibit limited lipotrophic properties. These physico-chemical features suggest that after its cleavage, the LukS-PV signal peptide could readily be released from the membrane translocon context to the cytosolic or extracellular aqueous environment. We thus hypothesized that at least some residues of this signal peptide bind either to the cell wall peptidoglycan or to its decorations, which include teichoic acids, polysaccharides and proteins, and modify the adhesive capacity of *S. aureus*. This is supported by the fact that the synthetic LukS-PV signal peptide, but not the scrambled synthetic peptide, when added externally to the bacteria, partially enhanced the adhesion property of LUG960. Similarly, the 2% SDS supernatant obtained from limited cell-surface extraction of the LukS-PV signal peptide positive strain was able to enhance the adhesive properties of LUG960. In contrast, cell-surface extracts prepared with mild detergents such as DM or DPC were not as efficient to transfer the adhesive capacity, suggesting that the LukS-PV signal peptide strongly binds to the cell wall. Indeed, the LukS-PV signal peptide was detected by western blot (using anti-histidine antibody) in the 2% SDS limited cell-surface extraction of the LukS-PV signal peptide positive strain (Figure 9). It is thus speculated that LukS-PV signal peptide behaves like Eap (extracellular adherence protein, also known as Map and p70), an adhesin with broad binding specificity but with particular affinity for the extracellular matrix and plasma proteins, which binds to the *S. aureus* cell wall by a sortase-independent mechanism. After translocation through the cytoplasmic membrane and even after being released into the extracellular space, Eap can re-associate to the *S. aureus* cell surface by means of several Eap-binding structures including the teichoic acids [24]. However, the limited efficiency of the synthetic signal peptide to confer increased adhesion in comparison to that produced *in vivo*, suggests that the peptide might act in conjunction with some other cell-wall components; moreover one cannot exclude that some post-translational modification of the signal peptide could occur in the physiological situation.

Mutagenesis of LukS-PV signal peptide and chimeras combining fragments of LukS-PV and LukE signal peptide lead to the hypothesis that the TSFHESKA sequence in the C-terminal half of LukS-PV-signal peptide is essential for association to the bacterial cell wall. Of note is that this sequence is unique to LukS-PV signal peptide and has no homology to any reported sequence in GeneBank. The cell wall determinant to which the LukS-PV signal peptide binds to remains to be determined. The fact that *Lactococcus lactis* expressing LukS-PV signal peptide showed enhanced adhesion suggests that the signal peptide-binding determinant could be likely the N-acetylglucosamine moiety or the wall-associated polymers, common features of Gram-positive bacteria. An alternative model to explain the enhanced attachment observed both when the LukS-PV signal peptide is produced by the *Staphylococcus* or when added externally, is that the signal peptide may bind and activate a surface receptor (such as a membrane histidine protein kinase) or, because of its small size and solubility it may diffuse in the cytosol, binds its cognate receptor and modulates (at the level of transcription or translation) the expression of proteins involved in adhesion. This model is in keeping with the extended properties of Gram-positive bacteria, which use small peptides as mating pheromones and quorum-sensing signals in bacterial communication systems [14]. Again, the fact that *Lactococcus lactis* strain expressing LukS-PV signal peptide displays enhanced attachment to ECM proteins renders the “signaling hypothesis” less likely and argues for a direct local effect on the cell surface.

Whatever the mechanism involved, the described binding features of the LukS-PV signal peptide to ECM components is not a general property of all other *S. aureus* signal peptides: the strains expressing LukF-PV signal peptide, TSST-1 signal peptide, LukD signal peptide and LukE signal peptide did not induce an enhanced adhesion phenotype. Both the mutagenesis experiments and the use of chimeric constructs reveal the strict requirement of three positives charges in the N-terminus for heparan sulfates binding to occur. The KKR N-terminal basic cluster of LukS-PV signal peptide constitutes a good candidate for heparan sulfates binding site [25]. Heparin and heparan sulfates belong to the family of glycosaminoglycans (GAGs) that consist in linear polysaccharides occurring in all animal cell surfaces and in the surrounding extracellular matrix generally as heparan sulfate proteoglycans [26]. The heparan sulfate glycosaminoglycans bind numerous adhesive and signaling molecules (cytokines, chemokines, growth factors, extracellular matrix proteins) thus working as a dynamic interface between the cells and the extracellular space [27], [28]. In addition, heparan sulfates bind efficiently to various extracellular matrix proteins and play an important role in mediating molecular bridges between the ECM proteins and between cells and ECM proteins through their heparin binding sites. This explains why purified ECM proteins may contain residual amounts of heparan sulfate chains as suggested by our data when using ECM proteins from commercial sources (Sigma-Aldrich Technical Service confirmed that they cannot guarantee the lack of residual heparan sulfates in their purified matrix protein preparations). This observation does not contradict the fact that *S. aureus* possesses specific and well characterized adhesins for ECM proteins [29]. But the fact that heparan sulfates, which are also highly abundant at the surface of respiratory epithelium, are the ligand of the LukS-PV signal peptide, fits well with the current model of necrotizing pneumonia [3], [5], [7] in which increased bacterial adhesion to the surface of the epithelium promotes bacterial infection. Note that other toxins from *S. aureus* also appear to target heparan sulfates of epithelial cells as beta-toxin increases the shedding of syndecan-1 (a major heparan sulfate proteoglycan), and syndecan-1 null mice are significantly protected from beta-toxin-induced lung injury [30].

Altogether, the present study provides new insights into the potential of signal peptides to contribute to other functions beyond protein secretion and propose an original mechanistic explanation of the enhanced adhesion of PVL positive strains to various ECM components.

## Materials and Methods

### Strains and growth conditions

*Escherichia coli* DH5α (Stratagene) and *S. aureus* RN4220, a nitrosoguanidine-induced mutant capable of accepting *E. coli* DNA [31], were used for plasmid amplification and genetic manipulations. *S. aureus* RN6390 derives from 8325-4 and is an standard *agr*+ strain. The phiSLTstrain (LUG855) was obtained by lysogenization of RN6390 with phiSLT phage carrying *luk*-PV [7], [32]. The phiSLTΔPVL strain (LUG776) is the partial deletion Δ*pvl ::tet*M mutant of *S. aureus* LUG855. In this strain, the PVL operon was not totally deleted; the first 41 amino acids LukS-PV and the last 128 amino acids of LukF-PV remained [32]. *S. aureus* strains LAC (USA300), LACΔPVL, MW2 (USA400) and MW2ΔPVL were constructed and generously provided by Dr. Frank DeLeo. Clinical1 is a *luk*-PV negative clinical strain from gut; clinical1phiSLT is clinical1 lysogenized with phiSLT; clinical2 is a *luk*-PV negative clinical strain from a furuncle; clinical2phiSLT is clinical2 lysogenized with phiSLT; clinical3 is a *luk*-PV negative clinical strain from pneumonia; clinical3phiSLT is clinical3 lysogenized with phiSLT. *Lactococcus lactis* subsp. *cremoris* 1363 and its derivative carrying a Luk-PV plasmid were generously provided by Dr Philippe Moreillon and Mathilde Ythier. The Luk-PV plasmid was made by insertion of the luk-PV operon onto the *L. lactis* vector pOri23 [33]. LukS-PV, LukS-PV signal peptide and LukE signal peptide were amplified and cloned on pOri 23 (Tables 1 and 2).

**Table 1:** Strains.

*S. aureus* strains	Relevant characteristics	Name used in text	Reference or source
8325-4	NCTC8325 cured of three prophages		[44]
RN4220	restriction-mutant of 8325-4		[31]
RN6390	derivative of 8325-4, *agr* positive		[45]
LUG855	RN6390phiSLT	RN6390phiSLT	[7], [32]
LUG776	LUG855 partial ΔPVL::*tmn*		
LUG960	RN6390Δ*spa*::kana	parental	This work
LUG1500	LUG776Δ*spa*::kana	chromosomal partial ΔPVL	This work
LUG1501	LUG960/pLUG534	Luk-PV plasmid	This work
LUG1137	LUG960/pLUG549	41aa Nterm LukS-PV	This work
LUG1155	LUG960/pLUG560	no insert plasmid	This work
LUG1157	LUG960/pLUG652	128aa Cterm LukF-PV	This work
LUG1159	LUG960/pLUG654	LukS-PV SP	This work
LUG1198	LUG960/pLUG670	LukS-PV ΔSP	This work
LUG1211	LUG960/pLUG676	TSST-1 SP	This work
LUG1225	LUG960/pLUG671	LukF-PV SP	This work
LUG1237	LUG960/pLUG725	LukF-PV plasmid	This work
LUG1238	LUG960/pLUG726	LukS-PV plasmid	This work
LUG1509	LUG960/pLUG732	LukE-SP	This work
LUG1510	LUG960/pLUG733	LukD-SP	This work
LUG1502	LUG960/ pLUG737	*luk*-PV mRNA	This work
LUG1503	LUG960/pLUG755	R5K	This work
LUG1504	LUG960/pLUG760	H24Q	This work
LUG1417	LUG960/pLUG774	H24A	This work
LUG1419	LUG960/pLUG775	S22P	This work
LUG1422	LUG960/pLUG776	1–25	This work
LUG1428	LUG960/pLUG777	1–22	This work
LUG1431	LUG960/pLUG778	K27A	This work
LUG1461	LUG960/pLUG786	K4N	This work
LUG1463	LUG960/pLUG787	K4N/K5A	This work
LUG1465	LUG960/pLUG788	K3A/K4N	This work
LUG1487	LUG960/pLUG797	LukS::LukE	This work
LUG1488	LUG960/pLUG798	E25Q	This work
LUG1493	LUG960/pLUG799	LukE::LukS	This work
LUG1631	LUG960/pLUG907	K3A/K4N::LukS-PV	This work
LUG1634	LUG960/pLUG908	His-LukS-PV	This work
LUG1637	LUG960/pLUG910	LukE::LukS::LukS-PV	This work
LUG1638	LUG960/pLUG911	K4N/K5A::LukS-PV	This work
LUG1642	LUG960/pLUG912	His-LukS-PV SP	This work
LUG1643	LUG960/pLUG913	LukS::LukE::LukS-PV	This work
LUG1601	*Lactococcus lactis* 1363/pLUG886	L.lactis LukS-PV	This work
LUG1602	*Lactococcus lactis* 1363/pLUG887	L.lactis LukE-PV SP	This work
LUG1616	*Lactococcus lactis* 1363/pLUG888	L.lactis LukS-PV SP	This work

**Table 2:** Plasmids.

Staphylococcal plasmids		
pC194	3 kb *S. aureus* plasmid, inducible chloramphenicol resistance (*cat*)	[46]
pSK265	pC194::pUC19 MCS	[36]
pLUG534	pSK265::*luk*-PV	This work
pLUG549	pSK265::Nter LukS-PV (41aa)	This work
pLUG560	pSK265::P-*luk*-PV–TT-*luk*-PV (PVL vector)	This work
pLUG652	pSK265::Cter LukF-PV (128aa)	This work
pLUG654	pSK265::LukS-PV signal peptide	This work
pLUG670	pSK265::LukS-PVΔ signal peptide	This work
pLUG671	pSK265::LukF-PV signal peptide	This work
pLUG676	pSK265::TSST-1 signal peptide	This work
pLUG725	pSK265::*luk*F-PV	This work
pLUG726	pSK265::*luk*S-PV	This work
pLUG732	pSK265::LukE signal peptide	This work
pLUG733	pSK265::LukD signal peptide	This work
pLUG737	pSK265::*luk*-PV mRNA	This work
pLUG755	pSK265::mutated LukS-PV signal peptide: R5K	This work
pLUG760	pSK265::mutated LukS-PV signal peptide: H24Q	This work
pLUG774	pSK265::mutated LukS-PV signal peptide: H24A	This work
pLUG775	pSK265::mutated LukS-PV signal peptide: S22P	This work
pLUG776	pSK265::shortened LukS-PV signal peptide: [1]–[25]	This work
pLUG777	pSK265::shortened LukS-PV signal peptide: [1]–[22]	This work
pLUG778	pSK265::mutated LukS-PV signal peptide: K27A	This work
pLUG786	pSK265::mutated LukS-PV signal peptide: K4N	This work
pLUG787	pSK265::mutated LukS-PV signal peptide: K4N/K5A	This work
pLUG788	pSK265::mutated LukS-PV signal peptide: K3A/K4N	This work
pLUG797	pSK265::chimeric signal peptide: LukS-PV [aa 1–20]::LukE [aa 21–28]	This work
pLUG798	pSK265::mutated LukS-PV signal peptide: E25Q	This work
pLUG799	pSK265::chimeric signal peptide: LukE [aa 1–20]::LukS-PV [aa 21–28]	This work
pLUG886	pORI23::LukS-PV signal peptide	This work
pLUG887	pORI23::LukE signal peptide	This work
pLUG888	pORI23::LukS-PV	This work
pLUG907	pSK265::mutated LukS-PV signal peptide K3A/K4N::LukS-PV	This work
pLUG908	pSK265::6xHis::LukS-PV	This work
pLUG910	pSK265::chimeric signal peptide: LukE [aa 1–20]::LukS-PV [aa 21–28]::LukS-PV	This work
pLUG911	pSK265::mutated LukS-PV signal peptide K4N/K5A::LukS-PV	This work
pLUG912	pSK265::6xHis::LukS-PV signal peptide	This work
pLUG913	pSK265::chimeric signal peptide: LukS-PV [aa 1–20]::LukE [aa 21–28]::LukS-PV	This work

*Staphylococci* were grown either on BM agar plates (1% peptone, 0.5% yeast extract, 0.1% glucose, 0.5% NaCl, 0.1% K2HPO4), or in brain-heart infusion (BHI), with chloramphenicol (20 µg/ml) or erythomycin (5 µg/ml) when appropriate. *L. lactis cremoris* was grown at 30°C in M17 medium (Oxoid) supplemented with 0.5% glucose [33].

### DNA manipulation and genetic constructions

Total DNA and plasmid DNA were prepared with standard methods [34]. Transformation of *Escherichia coli* DH5α and M15 was performed by treatment with CaCl_2_, and *S. aureus* strains were transformed by electroporation (Bio-Rad gene pulser).

Lysogenization was performed using 50 µl of phiSLT phage carrying *luk*-PV (10^11^–10^12^ UFP/ml) [32]. The deletion/replacement Δ*spa::kana* mutant of *S. aureus* RN6390 and LUG776 were obtained by using pMAD, a thermosensitive plasmid which contains a constitutively expressed ß-galactosidase gene that allows positive selection of double cross-overs by screening the ß-galactosidase activity on X-gal agar plates [35]. A 1 kbp DNA fragment corresponding to the kanamycin resistance gene was amplified from pCR2.1™ plasmid (Invitrogen) using primers kcr5/kcr6 and cloned between two DNA fragments corresponding to the flanking regions of protein A gene (*spa*) generated using oligonucleotides spa28/spa692 (664 pb) and spa2014/spa 2510 (496 pb), in pMAD. The resulting plasmid, pLUG196, was electroporated into RN4220, and then into RN6390 and LUG776. Transformants were grown at the non-permissive temperature (37°C), to select for cells in which the plasmid had been integrated into the chromosome by homologous recombination. To favor the second recombination event, a single colony was grown at 30°C for 10 generations and plated at 37°C overnight. Cells, which have lost the plasmid vector through a double crossing over event, were detected on X*gal* agar plates. PCR amplifications were used to confirm the loss of most of the *spa* gene, which was replaced by the kanamycin resistance gene. The resulting strains were designated LUG960 and LUG1500 (Table 1).

The *luk*-PV genes were expressed in *Staphylococcus* RN6390 or LUG960 by using plasmid pSK265, which is a derivative of pC194 with a pUC18 polylinker region inserted at its unique *Hin*dIII site [36]. All cloning were first made in *E. coli* on pBluescript™ vector (Stratagene), verified by sequencing and subcloned onto pSK265 restricted by *Xba*I and *Kpn*I. The *luk*-PV operon (including promoter and transcriptional terminator) was amplified using primers phi262 and phi2815. The 3′ regions of the *luk*-PV operon, were first cloned on a plasmid that contained the P-*luk*-PV promoter region (amplified using primers phi262/phi758) and the 5′ regions, on a plasmid that contained the *luk*-PV transcriptional terminator (amplified using primers phi2648/phi2815). In order to express intramolecular sequences of *luk*-PV or signal peptides, the promoter and terminator regions were cloned onto pSK265 separated by a short cloning sequence containing the restriction sites *Eco*RV and *Bam*HI (Tables 1 and 2).

### Colorimetric adhesion assay to ECM components

Adherence to elastin from human lung (Sigma), type I, IV, V collagen (Sigma) and laminin (Sigma) was performed in ninety-six–well microtiter plates as described [5]. Preparation of purified collagens I and V from distinct sources have been performed in our laboratory. Collagen I was extracted by pepsin digestion in 0.5 M acetic acid, 0.2 M NaCl at 4°C for 20 hours from bovine fetal bones and was subsequently purified by repeated salt-fractionation as described [37] and recombinant V produced in human embryonic kidney cell line (293-HEK) cells and purified as described previously [38].

To test the effect of heparin/heparan sulfates on adhesion, increasing concentrations of heparin (Sigma) or heparan sulfates (Sigma) were added to the bacterial suspension, and incubated for 30 min at 37°C. After treatment, the suspension was washed with 200 µL PBS 3 times and used in the adhesion assay.

To test the effect of digestion of ECM proteins with heparinase, increasing concentrations of heparinase III (Sigma) were added to laminin, elastin and type V, IV, I collagens (Sigma) coated wells, and incubated for 1 h at room temperature. After treatment, the wells were washed with 200 µL PBS 3 times, blocked with 200 µL of 0.2% BSA in PBS at 4°C for 2 h, and used in the adhesion assay.

To analyze whether the adhesive properties of LukS-PV signal peptide-expressing bacteria could be mediated by cell wall associated material, a brief 2% SDS or DPC or DM cell extract was prepared as follows: following culture and centrifugation, the bacterial pellet of the donor strain was resuspended in 1 mL extraction buffer (10 mM Tris-HCl pH 7.0, 2% SDS or DPC or DM), heated at 95°C for 2 min, and then centrifuged at 13,000×g for 10 min [39]. The supernatant (pure or diluted in PBS) was used to resuspend the bacterial pellet of the recipient strain to be tested in the adhesion assay to ECM proteins.

Synthetic signal peptide of LukS-PV (MVKKRLLAATLSLGIITPIATSFHESKA), LukF-PV (MKKIVKSSVVTSIALLLLSNTVDA) and scrambled peptide of LukS-PV signal peptide (PGAVHIKSIKTRIELMSLAKLTSAFTAL), were synthesized in-house (by S. Gurussiddappa) and tested for complementation of LUG960 cells in the adhesion assay. Synthetic peptides were added to the bacterial suspension (100 µL/ well; 10^9^ CFU/mL in PBS) at 0.5 to 1000 µg/mL final concentration and the mixture was loaded into the microtiter plates containing ECM components as described [5]. Alternatively, synthetic peptide was added to non adherent bacterial cells and the mixture was subjected to 2% SDS extraction as described above. The resulting extract was used to resuspend the bacterial pellet of the recipient strain to be tested in the adhesion assay to ECM proteins.

### Quantification of Panton Valentine Leukocidin production by ELISA

The level of PVL production in the supernatant after 24 h of staphylococcal culture was determined in duplicate by using an ELISA method as described previously [40].

### Surface plasmon resonance based binding assay

A BIAcore 3000 system, CM4 sensor chip, amine coupling kit and HBS-P buffer (10 mM HEPES, 150 mM NaCl, 3 mM EDTA, 0.005% surfactant P20, pH 7.4) were from Biacore AB., Heparan sulfate was from Celsus, streptavidin from sigma and biotin-hydrazine from Pierce. Heparan sulfate (HS) was biotinylated at its reducing end as described [41], and immobilized on a streptavidin activated sensorchip. For this purpose, 0.2 M of N-ethyl-N′-(diethylaminopropyl)-carbodiimide (EDC) and 0.05 M of N-hydroxysuccinimide (NHS), were injected for ten minutes over two flow cells of a CM4 sensorchip after which streptavidin at 0.2 mg/ml in 10 mM sodium acetate buffer, pH 4.2, was injected for a further ten minutes. Remaining activated groups were blocked with 1 M ethanolamine, pH 8.5. Typically, this procedure allowed the coupling of 3000–3500 resonance units (RU) of streptavidin on both flow cells. Biotinylated HS (100 µg/ml) was then captured to a level of 250 RU on one surface, the other one being left untreated to served as negative control. For binding assays, LukS-PV signal peptide in HBS-P was simultaneously injected over both negative control and HS surfaces for 5 min at 25°C and 50 µl/min. The HS surface was regenerated with a 250 µl pulse of 2 M NaCl. Control sensorgrams were subtracted on line from HS sensorgrams, and the resulting binding curves were analyzed using the Biaeval 3.1 software.

### Circular dichroism (CD)

Far UV CD spectra were recorded on a Chirascan spectrometer (Applied Photophysics, UK) calibrated with 1S-(+)-10-camphorsulfonic acid. Measurements were carried out at 298 K in a 0.1-cm path length quartz cuvette (Hellma), with peptide concentrations ranging from 58 to 150 µM. Spectra were measured in a 180 nm to 260 nm wavelength range with a increment of 0.2 nm, bandpass of 0.5 nm and integration time of 1 s. Spectra were processed, baseline corrected, smoothed and converted with the Chirascan software. Secondary structure analysis was carried out on the DICHROWEB server [42] using the CDSSTR program [43]. CD experiments were performed on the platform ‘Production et Analyse de Protéines’ from the IFR 128 BioSciences Gerland - Lyon Sud.

## Supporting Information

Figure S1 Inhibition of adhesion to ECM components by heparan sulfates Isogenic strains of Staphylococcus aureus examined for their binding capacity to the indicated ECM proteins (Y-axis, absorbance reading at 540 nm) coated on 96-well plates, at concentrations of 5 µg/mL (collagen I-panel A, collagen V-panel B, elastin-panel C, laminin-panel D). Heparan sulfates were added at decreasing concentrations (X-axis) to the bacteria before the adhesion assay. Parental (LUG960) indicates the genetic background in which the genetic modifications were made; LukS-PV SP, parental carrying a plasmid encoding the LukS-PV signal peptide. The vertical lines indicate the standard deviations.
(2.44 MB TIF)

Table S1 Quantification of Panton Valentine Leukocidin production by ELISA
(0.03 MB DOC)
