# Supplementary figures and images for: The Signal Peptide of Staphylococcus aureus Panton Valentine Leukocidin LukS Component Mediates Increased Adhesion to Heparan Sulfates
Source: PLoS One. 2009 Apr 6;4(4):e5042. doi: 10.1371/journal.pone.0005042 (PMC2661369; doi:10.1371/journal.pone.0005042)

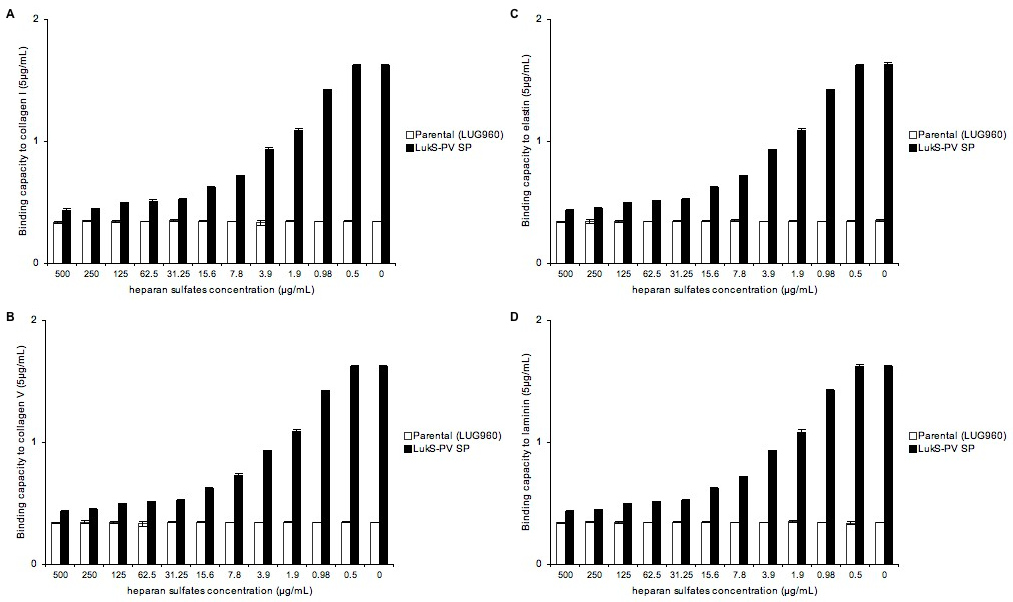

Supplement: Figure S1 — Inhibition of adhesion to ECM components by heparan sulfates Isogenic strains of Staphylococcus aureus examined for their binding capacity to the indicated ECM proteins (Y-axis, absorbance reading at 540 nm) coated on 96-well plates, at concentrations of 5 µg/mL (collagen I-panel A, collagen V-panel B, elastin-panel C, laminin-panel D). Heparan sulfates were added at decreasing concentrations (X-axis) to the bacteria before the adhesion assay. Parental (LUG960) indicates the genetic background in which the genetic modifications were made; LukS-PV SP, parental carrying a plasmid encoding the LukS-PV signal peptide. The vertical lines indicate the standard deviations. (2.44 MB TIF) [file pone.0005042.s001.tif]
