# Supplementary material for: The Signal Peptide of Staphylococcus aureus Panton Valentine Leukocidin LukS Component Mediates Increased Adhesion to Heparan Sulfates
Source: PLoS One. 2009 Apr 6;4(4):e5042. doi: 10.1371/journal.pone.0005042 (PMC2661369; doi:10.1371/journal.pone.0005042)
Supplement: Table S1 — Quantification of Panton Valentine Leukocidin production by ELISA (0.03 MB DOC) [file pone.0005042.s002.doc]

| **Strains** | **PVL levels (µg/mL) (mean±SD)** |
| --- | --- |
| LukS-PV plasmid | 41.75 ± 0.49 |
| His-LukS-PV | 36.05 ± 1.34 |
| K3A/K4N ::LukS-PV | 39.8 ± 0.57 |
| K4N/K5A ::LukS-PV | 39.2 ± 2.4 |
| LukS::LukE ::LukS-PV | 46.2 ± 0.57 |
| LukE::LukS ::LukS-PV | 34.7 ± 2.8 |
| LukS-PV ∆SP | 2.39 ± 0.04 |

The level of PVL production in the supernatant after 24h of culture was determined in duplicate by using an ELISA method as described previously [40].
